# Genetic diversity in two sibling species of the *Anopheles punctulatus *group of mosquitoes on Guadalcanal in the Solomon Islands

**DOI:** 10.1186/1471-2148-8-318

**Published:** 2008-11-24

**Authors:** Arif U Hasan, Setsuo Suguri, Chigusa Fujimoto, Rodney L Itaki, Masakazu Harada, Masato Kawabata, Hugo Bugoro, Bobogare Albino

**Affiliations:** 1 Department of International Medical Zoology, Faculty of Medicine, Kagawa University, 1750-1, Ikenobe, Miki, Kita, Kagawa, 761-0793, Japan; 2 Department of Medical Technology, Faculty of Health Sciences, Kagawa Prefectural College of Health Sciences, Hara, Mure, Takamatsu, Kagawa, 761-0123, Japan; 3 International Center for Medical Research, School of Medicine, Kobe University, Kusunoki, Chuo, Kobe, 650-0017, Japan; 4 Solomon Islands Medical Training and Research Institute, Honiara, the Solomon Islands

## Abstract

**Background:**

The mosquito *Anopheles irenicus*, a member of the *Anopheles punctulatus *group, is geographically restricted to Guadalcanal in the Solomon Islands. It shows remarkable morphological similarities to one of its sibling species, *An. farauti sensu stricto *(*An. farauti s.s*.), but is dissimilar in host and habitat preferences. To infer the genetic variations between these two species, we have analyzed mitochondrial *cytochrome oxidase subunit II *(*COII*) and nuclear ribosomal *internal transcribed spacer 2 *(*ITS2*) sequences from Guadalcanal and from one of its nearest neighbours, Malaita, in the Solomon Islands.

**Results:**

*An. farauti s.s*. was collected mostly from brackish water and by the human bait method on both islands, whereas *An. irenicus *was only collected from fresh water bodies on Guadalcanal Island. *An. irenicus *is distributed evenly with *An. farauti s.s*. (Φ_SC _= 0.033, 0.38%) and its range overlaps in three of the seven sampling sites. However, there is a significant population genetic structure between the species (Φ_CT _= 0.863, *P *< 0.01; Φ_ST _= 0.865, *P *< 0.01 and *F*_ST _= 0.878, *P *< 0.01). Phylogenetic analyses suggest that *An. irenicus *is a monophyletic species, not a hybrid, and is closely related to the *An. farauti s.s*. on Guadalcanal. The time estimator suggests that *An. irenicus *diverged from the ancestral *An. farauti s.s*. on Guadalcanal within 29,000 years before present (BP). *An. farauti s.s*. expanded much earlier on Malaita (*t*_exp _= 24,600 BP) than the populations on Guadalcanal (*t*_exp _= 16,800 BP for *An. farauti s.s*. and 14,000 BP for *An. irenicus*).

**Conclusion:**

These findings suggest that *An. irenicus *and *An. farauti s.s*. are monophyletic sister species living in sympatry, and their populations on Guadalcanal have recently expanded. Consequently, the findings further suggest that *An. irenicus *diverged from the ancestral *An. farauti s.s*. on Guadalcanal.

## Background

Extensive sampling and genetic studies have suggested that an endemic mosquito named *Anopheles irenicus *resides exclusively in the northern part of Guadalcanal Island (one of the Solomon Islands), along with *An. farauti sensu stricto *(*An. farauti s.s*.), *An. hinesorum*, *An. punctulatus *and *An. koliensis *[1,2]. All these mosquitoes are members of the *An. punctulatus *group, which was originally considered to comprise four closely related species, *An. farauti *Laveran, *An. punctulatus *Donitz, *An. koliensis *Owen and *An. clowi *Rozeboom & Knight ([3] and references therein). Further studies with cross-mating experiments, allozyme analysis and DNA probes finally revealed 12 sibling species within this *An. punctulatus *group: *An. farauti s.s*. Laveran (formerly *An. farauti *No. 1), *An. hinesorum *Schmidt (formerly *An. farauti *No. 2), *An. torresiensis *Schmidt (formerly *An. farauti *No. 3), *An. farauti *Nos. 4, 5, 6, *An. irenicus *Schmidt (formerly *An. farauti *No. 7), *An. punctulatus *Donitz, *An*. sp. near *punctulatus*, *An. koliensis *Owen, *An. rennellensis *Taylor & Maffi and *An. clowi *Rozeboom & Knight [3,4]. Nevertheless, the origin and population structure of the group remain obscure because of the involvement of these complex cryptic species [2,5].

A few striking differences between the endemic *An. irenicus *mosquito on Guadalcanal and one of its sibling species, *An. farauti s.s*., provide an excellent opportunity for investigating their genetic relationship. As mentioned earlier, *An. irenicus *is only found on Guadalcanal [1,2,4], but *An. farauti s.s*. is distributed from the east through New Guinea, the Bismarck Archipelago and the Solomon Islands to Vanuatu and southward into northern Australia [5,6]. Although these two species generally breed in similar types of water bodies such as small ground pools, margins of creeks, streams and even road ruts [1], they do not readily share their breeding sites. *An. farauti s.s*. almost always breeds in brackish water, whereas even though *An. irenicus *shows potential tolerance of brackish water, it is always found in breeding sites containing fresh water [7].

The adult mosquitoes also have distinct patterns of host dependence: *An. farauti s.s*. is anthropophilic but *An. irenicus *is zoophilic [1] and never bites humans [2]. Little is known about the reproduction of adult *An. irenicus*. In natural conditions adult *An. farauti s.s*. require a blood meal and oviposition usually occurs 48 to 52 hours later (Suguri et al., to be published elsewhere). Larval development in the laboratory is similarly irregular for both species, and delayed hatching of some eggs is common, resulting in the simultaneous occurrence of second instar larvae and pupae in the same rearing bowls (personal observation, [8]).

*An. farauti s.s*. and *An. irenicus *are morphologically nearly indistinguishable (like members of the *An. gambiae *complex [9]), but Schmidt et al. [4] described some subtle though definitive differences in morphological characters including the number of proepisternal setae in adults (*An. farauti s.s*. = 4 or more, *An. irenicus *= 3 or fewer), the number of branches of seta 5-V and seta 5-VI in pupae (*An. farauti s.s*. = 17 or fewer, *An. irenicus *= 18 or more), and the number of branches of seta 2-III in fourth instar larvae (*An. farauti s.s*. = 9 or fewer, *An. irenicus *= 10 or more). Schmidt *et al*. [4] therefore differentiated them taxonomically. However, some morphological variations were noted in the *An. farauti s.s*. collected from Australia, Papua New Guinea and the Solomon Islands [4]. Therefore, molecular analysis is necessary to identify and study them. Allozyme electrophoresis and mitochondrial DNA (mtDNA) sequences reveal distinguishable differences [2,5]. Moreover, Beebe *et al*. [1] showed species-specific ribosomal DNA polymorphisms by polymerase chain reaction-repeated fragment length analysis (PCR-RFLP) of ribosomal internal transcribed spacer (rITS) regions.

There have been previous studies of the distribution, habitat and morphology of *An. irenicus *in relation to *An. farauti s.s*., but less attention has been paid to their genetic variations on a finer scale. Therefore, we aimed to assess this issue using both mitochondrial (*cytochrome oxidase subunit II*; *COII*) and nuclear (ribosomal *internal transcribed spacer 2*; *ITS2*) markers. Mitochondrial DNA shows ample signatures of genomic events such as gene flow, migration, bottlenecks, speciation, hybridization and reinforcement [10-16], so it might give better information for studying current as well as historical genetic events affecting these mosquitoes [5,16]. On the other hand, comparison of the ITS region has clarified phylogenetic relationships among many closely-related species and in the *An. punctulatus *group [1], and will potentially make a substantial contribution to inferring the evolutionary relationships of *An. irenicus*.

Our previous work has added some insight about the phylogeography of *An. farauti s.s*. in Melanesia [17]. We have added further data from wider sampling areas of this species along with the endemic *An. irenicus *to shed light on genetic relationships between the species. In this study, therefore, we assessed the genetic variations between the species and particularly asked the following questions: (i) Is *An. irenicus *largely or completely sympatric with *An. farauti s.s*. on Guadalcanal? (ii) Are they monophyletic sister groups? (iii) Is there any demographic differences between them?

## Results

### Study samples

**Figure 1:**
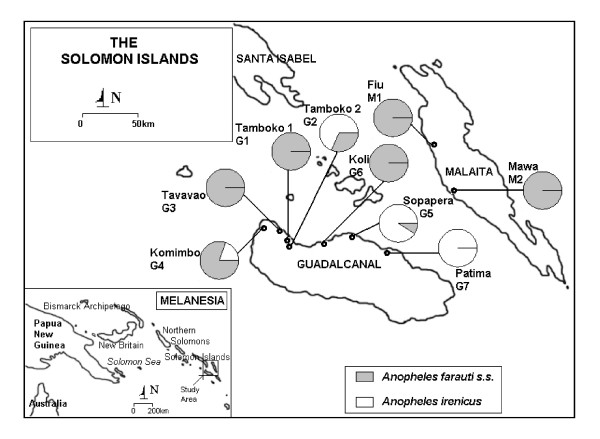
**Collection sites and frequencies of *Anopheles farauti s.s*. and *Anopheles irenicus *mosquitoes used in this study**. Dots indicate several nearer sampling sites. Pie charts represent the proportion of species collected from each site. Inset: The study area within Melanesia is shown.

Adult mosquitoes were collected from Tamboko 2 and Tavavao on Guadalcanal (Fig. 1 and details in [additional files 1 and 2]), and all of them were *An. farauti s.s*. (*n *= 51). We never collected *An. irenicus *on human baits. A total of 84 larvae were collected from 24 water bodies from five sites on Guadalcanal: Tamboko 2, Komimbo, Sopapera, Koli and Patima; and from two sites on Malaita: Fiu and Mawa (Fig. 1 and details in [additional files 1 and 2]). *An. irenicus *(*n *= 43) were only found in fresh water on Guadalcanal. The other 41 larvae were *An. farauti s.s*. and were mostly (*n *= 29, 70.7%) obtained from brackish water on Guadalcanal and Malaita. Only 12 *An. farauti s.s*. shared five out of the 20 water bodies with *An. irenicus *on Guadalcanal, and all five sites were fresh water bodies.

### Variability in sequences

The 684 bp *COII *gene was sequenced in all 92 *An. farauti s.s*. and 43 *An. irenicus *mosquitoes collected from the Solomon Islands. From these sequences, 26 Solomon Island-specific *An. farauti s.s*. haplotypes (S1 – S26, [additional file 2]) and 13 *An. irenicus *haplotypes (I1 – I13, [additional file 2]) were isolated (accession numbers in [additional file 1]), but no haplotypes were shared between the two species. Notably, the 12 *An. farauti s.s*. larvae collected from fresh water showed identical haplotypes (S1 and S2) to those collected from brackish water and by human bait. Among the 26 unique *An. farauti s.s*. haplotypes there were 22 variable sites (eight were parsimony-informative). Within the group, the range of uncorrected pairwise sequence divergence was 0% – 0.9%. *An. irenicus *exhibited less genetic variability than *An. farauti s.s*.: among 13 unique haplotypes, 12 variable sites with three parsimony-informative sites were found and the uncorrected pairwise sequence divergence ranged from 0% to 0.06%. The highest levels of haplotype diversity (0.82 ± 0.10) and nucleotide diversity (0.003 ± 0.002) were found in the Malaita population of *An. farauti s.s. An. farauti s.s*. on Guadalcanal showed only a 1.13-fold greater haplotype diversity (0.75 ± 0.04) than *An. irenicus *(0.66 ± 0.08), and the nucleotide diversity was the same (0.002 ± 0.001).

For *ITS2 *markers, 19 randomly-chosen *An. farauti s.s*. and seven *An. irenicus *samples were sequenced. All *An. farauti s.s*. and all *An. irenicus *sequences were identical, resulting in a single haplotype for each species (S1 and I1 for *An. farauti s.s*. and *An. irenicus*, respectively; accession numbers in [additional file 1]). A combined alignment of the two haplotypes consists of 563 bp, of which there are substitutions at eight sites and indels at 15 sites.

### Demographic analysis and divergence time

Significantly negative Tajima's *D *values were found only for the *COII *gene in the *An. farauti s.s*. (*D *= -1.94, *P *< 0.05) and *An. irenicus *(*D *= -1.69, *P *< 0.05) populations of Guadalcanal, suggesting a departure from neutral equilibrium (Table 1). These values were also interpreted in terms of demographic events and this revealed an excess of rare variants within these two populations. Strong evidence for rapid expansion of all three populations was provided by the significantly negative Fu's *Fs *values (*Fs *= -22.06, *P *< 0.001 and *Fs *= -3.81, *P *< 0.05 for *An. farauti s.s*. of Guadalcanal and Malaita, respectively; and *Fs *= -8.92, *P *< 0.001 for *An. irenicus*) (Table 1).

**Table 1:** Population parameters, neutrality tests and estimate of current demographic status estimated from *COII *data

Population	*Hd *± SE	π ± SE	*D*	*F*_S_	θ_f_	*N*_f_	*g*
*An. farauti s.s*.							
Guadalcanal	0.75 ± 0.04	0.002 ± 0.001	-1.94*	-22.06**	0.017	1.8 × 10^6^	957.5
Malaita	0.82 ± 0.10	0.003 ± 0.002	-1.44 ^*ns*^	-3.81 *	0.009	0.9 × 10^6^	859.1
*An. irenicus*							
Guadalcanal	0.66 ± 0.08	0.002 ± 0.001	-1.69*	-8.92**	0.013	1.4 × 10^6^	924.6

*Hd *and π are haplotype diversity and nucleotide diversity, respectively. *D *and *F*_S _are tests of neutrality; Tajima's *D *and Fu's *F*_S_, respectively. θ_f_, *N*_f _and *g *represent the current estimates of θ, current effective population size and growth rate estimated from LAMARC, respectively. ^*ns*^*P *> 0.05, *P ≤ 0.05 and **P ≤ 0.01

Both tests for validity (sum of square deviations, SSD, and raggedness index, RI) suggested that the mismatch distribution curves fitted significantly to the distribution under a model of population expansion (Table 2, see [additional file 3] for figures); however, there were clear differences in curve shape. The curve for *An. farauti s.s*. on Malaita was comparatively flat with a plateau, suggesting slower growth over a potentially longer time. In contrast, *An. farauti s.s*. on Guadalcanal showed a unimodal curve with a steeper leading face. This shape suggested a recent rapid population expansion after a bottleneck or after a founder effect from a small founder population. Interestingly, the *An. irenicus *population showed a unique L-shaped mismatch distribution, indicating recent demographic expansion with low molecular diversity (e.g. [11,18]). Although the shape of the mismatch distribution curve, particularly the slope of the leading face, can be influenced by sudden population expansion from equilibrium as well as by population bottlenecks, simulations have shown that stable populations almost never produce this type of shape [19]. Indeed, the estimates of time τ and the current and historical population parameters θ imply a historical episode of expansion of all three populations (τ > 0 and θ_1 _> θ_0_; Table 2). The probable times of initiation of population expansion (*t*_exp_) were about 24,600 BP (95% CI: 8,600 BP – 38,000 BP) on Malaita and about 16,800 BP (95% CI: 12,000 BP – 22,000 BP) on Guadalcanal for *An. farauti s.s*., and about 14,000 BP (95% CI: 4,700 BP – 27,000 BP) for *An. irenicus *(Table 2). Nevertheless, a similar type of mismatch distribution can occur when an initial population is restricted to a very small area and subsequently expands over time and space. The resulting population becomes genetically subdivided as individuals tend to mate only with geographic neighbours [20]. In the later part of this section we show that the extensive gene flow within islands was completely contrary to the population substructure and particularly the spatial expansion model of mismatch distribution.

**Table 2:** Parameters of the sudden expansion model estimated from COII data.

Population	τ	*t*_exp _BP	θ_0_	θ_1_	SSD	RI
*An. farauti s.s*.						
Guadalcanal	1.31 (0.81–1.93)	16,800 (12,000–22,000)	0 (0–0.03)	∞ (5.52-∞)	0.003 ^*ns*^	0.07 ^*ns*^
Malaita	1.922 (0.56–3.12)	24,600 (8,600–38,000)	0 (0–0.17)	26.28 (2.95-∞)	0.001 ^*ns*^	0.04 ^*ns*^
*An. irenicus*						
Guadalcanal	1.117 (0.31–2.58)	14,000 (4,700–27,000)	0 (0–0.33)	11.50 (1.16-∞)	0.006 ^*ns*^	0.03 ^*ns*^

τ, *t*_exp_, θ_0 _and θ_1 _are, respectively: expansion parameter, time of initiation of population expansion, and θ parameter before and after expansion. Values of 95% CI are within parentheses. SSD and RI are sum of squared deviations and raggedness index, respectively. ^*ns*^*P *> 0.05

Evidence for population expansion was also inferred from the high *g *values assessed from LAMARC (Table 1). Therefore, we estimated the present-day effective female population sizes (*N*_f_) from the maximum likelihood estimates of the current effective values of θ (θ_f_). The current effective female population sizes of *An. farauti s.s*. (1.8 × 10^6^) and *An. irenicus *(1.4 × 10^6^) on Guadalcanal were almost twice that of *An. farauti s.s*. on Malaita (0.9 × 10^6^).

The estimated time of divergence between *An. irenicus *and *An. farauti s.s*. produced by MDIV (*T*_div _= 1.28) was 32,800 BP. Regarding the *T*_MRCA_, we anticipated that all the haplotypes sampled coalesced 2.15 units ago, corresponding to 55,000 BP. However, the divergence time was not estimated from *ITS2 *because this marker system can be influenced by homogenization among different loci within multigene DNA families (i.e. concerted evolution [21]).

### Phylogenetic analysis

**Figure 2:**
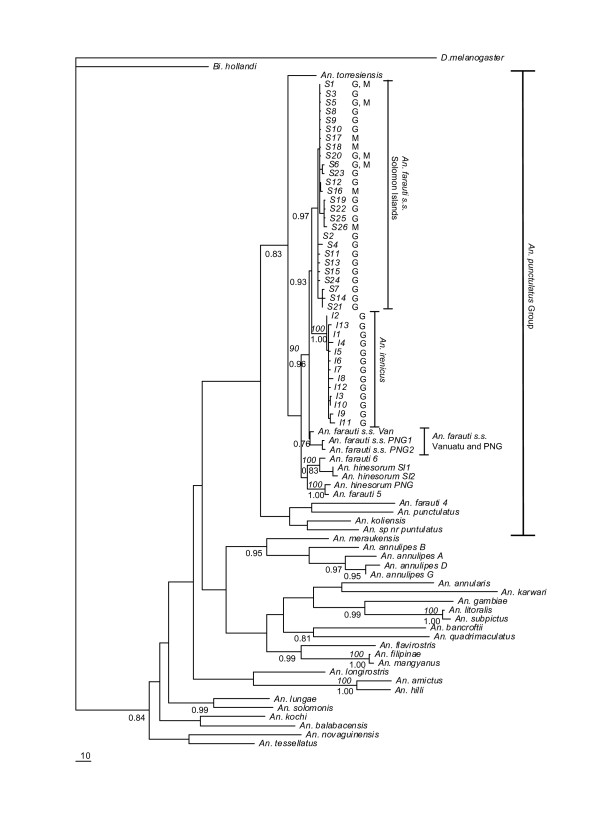
**Maximum Parsimony (MP) consensus tree for 684 bp *COII *haplotypes in mitochondrial DNA using the GTR+I+G model**. The trees were rooted with *D. melanogaster *and *Bi. hollandi*. Bootstrap values of > 90% are shown above the branch in italics and posterior probability values of > 0.75 are shown below the branch. *S *and *I *indicate haplotypes of *An. farauti s.s*. of the Solomon Islands and *An. irenicus*, respectively. Collection sites are represented after haplotypes where sampled from multiple sites. G, Guadalcanal; M, Malaita; *SI*, Solomon Islands; *PNG*, Papua New Guinea; *Van*, Vanuatu Islands. Sample codes and GenBank accession number for each specimen are given in [additional file 1].

We aligned all 26 *An. farauti s.s*. and 13 *An. irenicus *haplotypes obtained in this study along with 37 sequences from 32 commonly-occurring members of the genus *Anopheles *in this geographical region and two outgroups (*Bironella hollandi *and *Drosophila melanogaster*) for mitochondrial *COII*. The final alignment thus consisted of 687 bp. The phylogenetic trees constructed by the MP and Bayesian methods using these sequences were very similar in topology. Therefore, only a consensus MP tree topology is shown in Figure 2. Here, *An. farauti s.s*. haplotypes were found to be geographically highly structured and composed of two separate subgroups. In one subgroup, the two haplotypes of Papua New Guinea were grouped with the single haplotype of Vanuatu; in the other, all *An. farauti s.s*. haplotypes of the Solomon Islands were clustered together. *An. irenicus *formed a monophyletic clade and grouped with the *An. farauti s.s*. subgroup of the Solomon Islands with strong support (posterior probability = 0.93).

**Figure 3:**
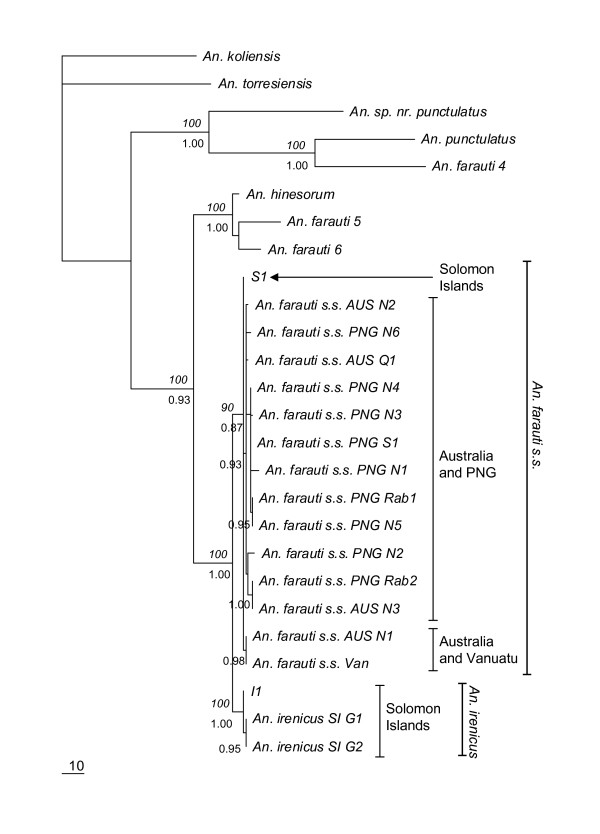
**Maximum Parsimony (MP) consensus tree for 760 bp *ITS2 *haplotypes in nuclear ribosomal DNA using the HKY85+G model**. The trees were rooted with *An. koliensis*. Bootstrap values of > 90% are shown above the branch in italics and posterior probability values of > 0.90 are shown below the branch. *S1 *and *I1 *indicate haplotypes of *An. farauti s.s*. of the Solomon Islands and *An. irenicus*, respectively. For species collected from multiple sites, species names are followed by localities. *AUS*, Australia; *PNG*, Papua New Guinea; *SI*, Solomon Islands; *Van*, the Vanuatu Islands; *G*, Guadalcanal; *N*, northern part; *S*, southern part; *Rab*, Rabaul; *Q*, Queensland. Sample codes and GenBank accession number for each specimen are given in [additional file 1].

It was not possible to include all species from the *COII *for the *ITS2 *because their representative sequences were not available in GenBank. Moreover, some sequences from different taxa were too divergent with respect to the *An. punctulatus *group to be aligned unambiguously. So the *ITS2 *dataset was finally constructed with 24 additional sequences (obtained from GenBank, [additional file 1]) representing the *An. punctulatus *group, rendering *An. koliensis *the only outgroup. The alignment of *ITS2 *sequences consisted of 760 bp. Trees based on MP and Bayesian approaches showed very similar topologies. The only exception was the position of *An. torresiensis*. In the topology obtained with the Bayesian approach, *An. torresiensis *appears to be a sister of a clade containing *An. farauti s.s*., *An. irenicus*, *An. hinesorum*, *An. farauti *5 and *An. farauti *6 (figure not shown), while in the MP trees it forms a tritomy involving the outgroup (*An. koliensis*) and a clade containing the remaining species (Figure 3). However, the relationship between *An. farauti s.s*. and *An. irenicus *was the same irrespective of the approach used. The trees for *ITS2 *were less robust than the *COII *tree in taxon richness but more informative about the extensive variation among geographical sampling sites for *An. farauti s.s*., and they resolved the relationships of this species more precisely. Unlike the *COII *phylogeny, the trees based on *ITS2 *sequences were inconsistent with paraphyly of *An. farauti s.s*. In *ITS2 *trees, *An. farauti s.s*. formed a monophyletic group comprising three monophyletic subgroups, each with high support (90% and 0.87 for bootstrap and posterior probability, respectively). The first *An. farauti s.s*. subgroup was composed of a single haplotype from the Solomon Islands. The second was composed of one northern Australian haplotype and the Vanuatu haplotype. The last major subgroup was composed of the remaining haplotypes found in Australia and all the haplotypes found in Papua New Guinea. *An. irenicus*, on the other hand, was well defined and formed a monophyletic clade. *An. irenicus *along with *An. farauti s.s*. formed a sister group to *An. hinesorum*, *An. farauti *5 and *An. farauti *6.

The phylogenetic relationships among some members of the *An. punctulatus *group within the Australasian region were not well resolved and showed many genetically distinct lineages restricted within different geographical ranges. A detailed discussion of this *Punctulatus *group will be published elsewhere.

### Population genetic structure and gene flow

Genetic differentiation between all pairs of geographical samples was estimated at each sampling site. Neither the *An. farauti s.s*. nor the *An. irenicus *population showed significant pairwise estimates of differentiation (*F*_ST_) within any of the islands (Table 3). As anticipated, however, the level of differentiation within *An. farauti s.s*. between the two islands was relatively high because of the sea barrier. In particular, Tamboko 1 (G1), Tamboko 2 (G2) and Tavavao (G3) on Guadalcanal were significantly different from Fiu (M1) and Mawa (M2) on Malaita. The corresponding gene migration (*Nm*) values revealed restricted gene flow between most of the sites on these two islands (Table 3). Only Sopapera (G5) showed extensive gene flow with the Malaita populations (M1 and M2). Differentiation between *An. farauti s.s*. and *An. irenicus *was also significant and gave better contrast when the samples were grouped together by island. Significant differences were found between the two *An. farauti s.s*. populations on Guadalcanal and Malaita (*F*_ST _= 0.132, *P *< 0.01; see [additional file 4]), and in accordance with expectation, both *An. farauti s.s*. populations differed significantly from the *An. irenicus *population (*F*_ST _= 0.885, *P *< 0.01; and 0.878, *P *< 0.01 from Guadalcanal and Malaita, respectively). Genetically, the *An. irenicus *population was also isolated from both *An. farauti s.s*. populations; interspecific gene migration is strongly restricted (*Nm *= 0.065 and 0.069 for Guadalcanal and Malaita, respectively).

**Table 3:** Pairwise genetic distances and migration rates based on each site estimated from *COII *data

	*A. f*. (G1)	*A. f*. (G2)	*A. f*. (G3)	*A. f*. (G4)	*A. f*. (G5)	*A. f*. (G6)	*A. f*. (M1)	*A. f*. (M2)	*A. i*. (G2)	*A. i*. (G4)	*A. i*. (G5)
*A. f*. (G1)		∞	∞	19.43	∞	∞	4.35	4.12	0.06	0.07	0.07
*A. f*. (G2)	-0.02		∞	∞	35.32	∞	2.64	1.87	0.04	0.05	0.07
*A. f*. (G3)	-0.01	-0.06		59.26	12.32	∞	1.83	1.48	0.04	0.05	0.06
*A. f*. (G4)	0.03	-0.02	0.01		∞	∞	5.01	5.73	0.06	0.11	0.09
*A. f*. (G5)	-0.20	0.01	0.04	-0.13		12.17	∞	∞	0.04	0.02	0.08
*A. f*. (G6)	-0.01	-0.05	-0.03	-0.09	0.04		2.41	2.47	0.04	0.06	0.08
*A. f*. (M1)	0.10*	0.16*	0.22‡	0.09*	-0.25	0.17*		9.55	0.05	0.09	0.08
*A. f*. (M2)	0.11*	0.21‡	0.25‡	0.08	-0.22	0.17	0.05		0.05	0.07	0.08
*A. i*. (G2)	0.90‡	0.93‡	0.93‡	0.89‡	0.93‡	0.92‡	0.91‡	0.92‡		75.83	5.60
*A. i*. (G4)	0.88‡	0.91‡	0.92‡	0.82‡	0.96	0.88*	0.86‡	0.88‡	0.01		∞
*A. i*. (G5)	0.87‡	0.87‡	0.89‡	0.84‡	0.86‡	0.86‡	0.86‡	0.86‡	0.08‡	-0.01	

Pairwise *F*_ST _values, below diagonal; pairwise *Nm *values, above diagonal. *A. f*. and *A. i*. are *An. farauti s.s*. and *An. irenicus*, respectively. G1, Tamboko 1; G2, Tamboko 2; G3, Tavavao; G4, Komimbo; G5, Sopapera; G6, Koli; M1, Fiu; M2, Mawa. Patima (G7) was not included because only a single haplotype was collected from this site.

*P ≤ 0.05 and ‡*P *≤ 0.01

Our AMOVA findings were also consistent with the pairwise *F*_ST _values for the combined data [additional file 5]. The *An. farauti s.s*. populations on Guadalcanal and Malaita and the *An. irenicus *population differed significantly from each other (Φ_CT _= 0.863) and were responsible for 86.26% of the genetic variance. Haplotypes within these three populations related to the total samples were also significantly different (Φ_ST _= 0.865) and explained most of the remaining (13.55%) differences. However, variation among geographical ranges within each of the three populations was almost negligible (Φ_SC _= 0.014) and could not explain any of the variation adequately (0.20%). Subsequently, AMOVA for Guadalcanal also showed significant population structure between *An. farauti s.s*. and *An. irenicus *(Φ_CT _= 0.886, 88.57% and Φ_ST _= 0.889, 11.06%) and almost no variation among geographical ranges within these two species (Φ_SC _= 0.033, 0.38%) [additional file 5].

## Discussion

### Sympatric distribution

Sometimes, sampling from insufficient geographical sites can lead to an erroneous inference of sympatric distributions of species that are actually more widely distributed and are sympatric only over a small part of their distribution ([22] but see also [10]). We collected *An. irenicus *from four of the seven sites at which it is known to occur on Guadalcanal. On the other hand, *An. farauti s.s*. was isolated from six sites on Guadalcanal (Tamboko 1, Tamboko 2, Tavavao, Komimbo, Koli and Sopapera). However, despite the failure to collect *An. farauti s.s*. from Patima and *An. irenicus *from Tamboko 1, Tavavao and Koli, which might indicate an error in sampling site choice, they apparently cover most of the north of Guadalcanal (Fig. 1) and thus these two species are distributed in complete sympatry within the island.

The sympatric distribution of *An. farauti s.s*. with *An. irenicus *on Guadalcanal is also apparent from the non-significant pairwise population differences (*F*_ST_) in the *COII *sequences of their respective clusters, which simultaneously excludes geographical structure in either *An. farauti s.s*. or *An. irenicus*. Gene flow and the corresponding migration rate within the Guadalcanal populations of *An. farauti s.s*. were extremely high. Although Patima was excluded because of the small sample size, a similar pattern – absence of gene flow barrier with moderate to high migration rate – was also apparent in the *An. irenicus *populations. The AMOVA further detected the homogenous distribution of the two species, with only 0.38% molecular variance between the localities sampled on Guadalcanal [additional file 5]. Similarly, the wide distribution of the single *ITS2 *haplotype for both *An. farauti s.s*. and *An. irenicus *also implied the absence of a gene flow barrier within the Guadalcanal populations.

Notably, populations of *An. farauti s.s*. were highly differentiated at the *COII *locus between Guadalcanal and Malaita; however, the Sopapera population on Guadalcanal was not significantly differentiated from populations on Malaita (Table 3). It is possible that gene flow between populations on the two islands occurs across the sea gap, with Sopapera representing the nearest population receiving immigrants from Malaita. AMOVA showed that *An. irenicus *and *An. farauti s.s*. on these two islands were significantly different among groups (Φ_CT _= 0.863, 86.26%) as well as among populations within a group (Φ_ST _= 0.865, 13.55%), and revealed that gene flow between the islands was restricted ([additional file 5], also in [17]). Moreover, it is obvious that the sea gap must have prevented the dispersal of *An. irenicus *from Guadalcanal to Malaita (see also [23-26]). Rather, the simplest explanation of the apparent inter-island gene flow is that the haplotypes isolated from the two Sopapera samples were both S1, which is the commonest haplotype within this region. Therefore, the presence of a single shared haplotype (S1) has nullified the genetic differentiation and no difference between the populations is apparent [additional file 2], whereas both the Guadalcanal and Malaita populations are genetically isolated *per se *[17].

### Sister taxa

The assumption of sister taxa is essentially based on two attributes: it must reflect true phylogenetic status and not the genetic similarity between more distant species that can result from hybridization [16]. Phylogeny using mitochondrial *COII *data showed that *An. farauti s.s*. of the Solomon Islands exhibits significant differentiation from all *An. farauti s.s*. in other geographical regions and forms a monophyletic assemblage with *An. irenicus*. Our previous study showed that the genetically divergent Solomon Islands subgroup of *An. farauti s.s*. arose because of repeated bottlenecks and lineage sorting for adaptation during the dispersion from Papua New Guinea [17]. Therefore, the topology reveals that *An. irenicus *and *An. farauti s.s*. truly share a common ancestry.

The sub-structuring in the phylogeny of *An. farauti s.s*. implied by mtDNA is well resolved in nuclear rDNA *ITS2 *trees, and reveals that *An. farauti s.s*. and *An. irenicus *are sister taxa. However, the haplotypes of *An. farauti s.s*. collapsed into a tritomy: the Solomon Islands subgroup, the Australia-Vanuatu subgroup and the Australia-Papua New Guinea subgroup. Because of the presence of this tritomy within different geographical locations, the position of the Solomon Islands haplotype in relation to other haplotypes could not be detected. However, a plausibly close relationship between *An. farauti s.s*. and *An. irenicus *on the Solomon Islands can be inferred as implied by the *COII *analysis, which may indicate genealogical congruence and thus can be interpreted in terms of a common origin for both species.

Regarding the second attribute (hybridization) for assuming sister taxa, a small amount of introgression or hybridization can homogenize mtDNA and make species appear closely related [16]. The Solomon Islands are situated between Papua New Guinea and Vanuatu (Fig. 1), so the *An. farauti s.s*. of either Papua New Guinea or Vanuatu may have originated allopatrically, made secondary contact with the Solomon Islands population and generated a hybrid species (i.e. *An. irenicus*). According to this hybridization hypothesis, there will be no prezygotic isolation between *An. farauti s.s*. clades [16], so putative hybrids could be formed and viable hybrid populations would be genetically very similar to the parental *An. farauti s.s*. However, in this study no *An. irenicus *was retained with the *An. farauti s.s*. of Papua New Guinea or Vanuatu (*F*_ST _= 0.918 and 0.902 with Papua New Guinea and Vanuatu, respectively; Table 4). Therefore, the monophyly shown by the phylogenetic trees suggests that *An. irenicus *is truly a near-sister species of *An. farauti s.s*. and not a putative hybrid [2,4] that diverged from the ancestral *An. farauti s.s*. on Guadalcanal.

### Divergence and expansion

Population history revealed that *An. farauti s.s*. dispersed from Papua New Guinea to the Solomon Islands concomitantly with human settlement during the recent Pleistocene (c. 29,000 BP; [17,27]). Therefore, acknowledging the uncertainties in predicting divergence time [28], we propose that it is unlikely that *An. irenicus *diverged from *An. farauti s.s*. prior to 29,000 BP (corresponding to 348,000 generations, assuming 12 generations per year [17]). Indeed, this time is sufficient to develop the genetic variation required to evolve a new species under the influence of selection, because this process includes an unstable intermediate stage that must be traversed rapidly [11,16,29,30]. It is beyond the scope of the current study to determine the mode of speciation of *An. irenicus*; further work is needed to resolve the issue.

A demographic expansion of the mosquito population is expected at approximately the same time as the host (human) population expansion [13]. For *An. farauti s.s*. on Malaita, demographic analyses indicate the population expansion occurred around 24,600 BP (Table 2) and this is somewhat after the first human settlement in the Solomon Islands (c. 29,000 BP; [27,31]). In contrast, a delayed but time-synchronized single expansion was observed for both species on Guadalcanal (16,800 BP and 14,000 BP for *An. farauti s.s*. and *An. irenicus*, respectively). The delay in population expansion on Guadalcanal might have resulted from a substantially low human (host) population on Guadalcanal. Consistent with these assumptions, anthropological history suggests that only Malaita had sizeable human populations. The other islands including Guadalcanal were inhabited by very few humans [23], and after the Last Glacial Maximum (LGM) human expansion occurred in the region around 3,500 to 8,000 BP [31]. Although the human (host) population size can potentially explain the population expansion of *An. farauti s.s*. on both islands, the population expansion of *An irenicus *cannot be unambiguously explained as no census is available for its host (i.e. birds or animals), and it requires further assessment. Nevertheless, from the maximum likelihood estimates of θ_f_, (Table 1) the effective population sizes, *N*_f_, of *An. irenicus *and *An. farauti s.s*. on Guadalcanal were also twice as large as that of *An. farauti s.s*. on Malaita. In this context, we estimate the timeframe defining the initiation of divergence of *An. irenicus *from *An. farauti s.s*. (c. 29,000 BP) to its final expansion (*t*_exp _= 14,000 BP) to occur during the LGM. During the LGM the sea level fell to its minimum level, which made breeding in brackish coastal regions much more difficult. It can be anticipated that unavailability of favourable breeding sites may have driven some of the ancestors to adapt to an alternative niche (i.e. large fresh water bodies), readily available only on Guadalcanal [23]. During the non-arid deglaciation following the LGM (c. 17,000 BP [32]) both the sea level and the fresh water content gradually started to rise, favouring both the species that continued to oviposit on brackish water and those that started to oviposit on fresh water, and a post-LGM population expansion occurred on Guadalcanal (*t*_exp _= 16,800 BP and 14,000 BP for *An. farauti s.s*. and *A. irenicus*, respectively). Considering all the aforementioned scenarios, it is not unreasonable to propose that a greater variety of new hosts and exposure to novel breeding sites may explain this larger value, notwithstanding similar growth rates (Table 1).

## Conclusion

These findings suggest that *An. irenicus *and *An. farauti s.s*. are monophyletic sister species living in sympatry, and their populations on Guadalcanal have recently expanded. Consequently, the findings further indicate that *An. irenicus *diverged from the ancestral *An. farauti s.s*. However, knowledge of the number of genes involved in oviposition site and host choice, mating behaviour and the anthropogenic history of this region, as well as a more comprehensive approach using additional genetic markers such as microsatellites and amplified fragment length polymorphism together with wider geographical sampling, are necessary to ascertain the origin of *An. irenicus *on this particular island (e.g. [10,11,30,33,34]).

## Methods

### Sampling

We collected mosquitoes from Guadalcanal and from one of its nearest neighbours among the Solomon Islands, Malaita (Figure 1, details in [additional files 1 and 2]). Mosquitoes from Malaita were sampled to achieve confidence in sister lineage assessment (e.g. [10,11,22]); to date, no evidence of occurrence of *An. irenicus *has been found on Malaita ([2], personal observation). Per annum rainfalls in Guadalcanal and Malaita are about 2000 mm and 3000 mm, respectively. During June to September the climate is comparatively dry in Guadalcanal (c. 92 mm per month), but in Malaita the rainfall is rarely below c. 190 mm per month. Both adults and larvae of *An. farauti s.s*. as well as *An. irenicus *larvae were caught abundantly during the wet season. The number of *An. irenicus *larvae declined more than those of *An. farauti s.s*. during the dry seasons. This was due to the drying up of fresh water habitats, whereas the brackish seawater pools remained almost unchanged, though breeding sites were occasionally washed out after heavy rainfall in both the wet and dry seasons. Therefore, mosquitoes were first collected during the wet season (January to February of 2005) and subsequently during the drier seasons (July to September of 2005 to 2007) when the condition of the breeding sites remained stable. A total of 20 water bodies from five sites on Guadalcanal, Tamboko 2 (G2), Komimbo (G4), Sopapera (G5), Koli (G6) and Patima (G7), and four from two sites on Malaita, Fiu (M1) and Mawa (M2) are described here. To avoid sampling bias, only third and fourth instar larvae were collected from at least two (maximum eight) water bodies at each site using the standard larval dipper method [35]. Each water body was scooped by five collectors at different spots for about 5 minutes, less if larval densities were high. These sampling sites included fresh water bodies such as streams, ponds, ground pools and swamps, and brackish water bodies such as edges of creeks and water flooding the beach. Sampling was from multiple sites because siblings can result at any one breeding site owing to the oviposition of a single female. For the same reason, sites situated at a minimum distance of 300 meters were selected for sampling. Adult mosquitoes were collected by the human bait method [12] from Tamboko 1 and Tavavao (sites marked G1 and G3, respectively in Figure 1). Field materials were first screened morphologically as *An. farauti s.l*. [4,23,36]. They were then sealed in individual gelatin capsules, given unique identification numbers and preserved with desiccant at room temperature [12].

### Amplification and sequencing

Total genomic DNA was extracted using a QIAamp DNA Minikit (Qiagen, K.K. Japan) following the manufacturer's instructions. Mitochondrial *COII *was amplified with the *An. punctulatus *sibling species-specific primers *COII *A [5] and *COII *H (5' -CCAATTAATAGGGGCTATTTGTGGG-3') in an ASTEC PC-320 thermocycler (ASTEC- Human Science, Japan). This sequence includes ATG for initiation and excludes a T-tRNA for termination [5]. A fragment of the nuclear ribosomal *ITS2 *region was amplified using the primers *ITS2*A and *ITS2*B described by Beebe & Saul [37]. In both cases the PCR products were purified on a 5% acrylamaide gel [38] and cycle-sequenced in both directions with corresponding PCR primers using a big dye terminator sequencing kit (Applied Biosystems). Sequences were run on an ABI prism™ 377 DNA Sequencer (Applied Biosystems). Forward and reverse strands were assembled for each individual sample in the SEQMANII version 3.6.0 (DNASTAR, Inc.) sequence editor program and a single sequence was defined.

### Variability in sequences

All sequences were aligned using MEGA version 3.1 [39]. No length differences were found in the *COII *alignment. The amino acid sequences were then inferred using the *Drosophila *mtDNA genetic code in DNASP version 4.10 [40] to check for the presence of ambiguous stop codons. For comparison, we randomly chose 19 *An. farauti s.s*. and seven *An. irenicus *samples identified from *COII *sequencing. These samples were analyzed for *ITS2 *and included in the study. The position and length of the *ITS2 *sequence were also determined by comparison with published DNA sequences from GenBank.

Basic sequence statistics and various population parameters were computed using DNASP and MEGA. Genetic diversity at the intra-population level was measured in terms of haplotype diversity (*Hd*, [41]) and nucleotide diversity (π, [41]). Haplotypes were identified and their relative frequencies within populations were calculated using ARLEQUIN version 3.1 [42]. For some tests we pooled each species according to geographical origin to obtain better-compiled values. Some data were reproduced with permission.

### Demographic analysis and divergence time

Selective neutrality was assessed by Tajima's *D *[43] and Fu's *F*_S _[44] tests. Estimators related to population expansion, τ (age of expansion), θ_0 _and θ_1 _(θ before and after expansion, respectively), were computed with 95% CI in ARLEQUIN. The time of the main expansion (*t*_exp_) was estimated from the equation τ = 2*ût*_exp _(*û *is the substitution rate of the entire sequence estimated from *û *= *m*_T_μ; here, *m*_T _is the number of nucleotides in the sequence under study and μ = 0.057 substitutions per site per million years [45,46]).

The observed number of differences between pairs of haplotypes was plotted to obtain a mismatch distribution. A population that has passed through a recent demographic expansion shows a unimodal distribution, whereas a population at equilibrium gives a multimodal distribution [19,47]. The validity of the estimated expansion model is tested by the distribution of the sum of square deviations (SSD) between observed and expected values obtained by the parametric bootstrap approach [48]. A significant SSD value is taken as evidence for departure from the estimated demographic model, which can be a model of either population expansion (if τ > 0 and θ_1 _> θ_0_) or a stationary population (if τ = 0 and θ_1 _= θ_0_). In addition, we computed the raggedness index (RI) of the observed distribution [49]. This index takes larger values for the multimodal distributions typical of a stationary population than for the unimodal and smoother distributions typical of expanding populations. Significance is then tested by the parametric bootstrap approach mentioned earlier.

We used LAMARC version 2.0.2 [50] to estimate the current demographic status of *An. farauti s.s*. and *An. irenicus *populations. The model and parameters of substitution were obtained from MODELTEST (described in next section). LAMARC uses Markov chain Monte Carlo (MCMC) sampling and takes both historical and asymmetric gene flow into account. Our estimates included the current effective population size *N*_f _(from θ_f _= 2*N*_fμ_) and growth rate (*g*) under the exponential growth model. The parameters θ_f _and μ represent the current estimates of θ and the neutral mutation rate per site per generation, respectively. This program was run for 10 initial chains of 10,000 steps and two final chains of 200,000 steps. To improve accuracy, we used four steps at different temperatures; one cold and three hot searches.

We used the program MDIV ([51], http://cbsuapps.tc.cornell.edu/mdiv.aspx) to determine divergence time (*T*_div _= *t*_1_*u*) and the expected time of the most recent common ancestor (*T*_MRCA _= *t*_2_*u*) for all sequences in the samples by the MCMC method within a likelihood framework using the finite site model (Hasegawa, Kishino, Yano [HKY], [52]). Here *u *is the mutation rate per sequence per year, and *t*_1 _and *t*_2 _are, respectively, the population divergence time in years before the present (BP) and the gene coalescence time in years before the present (BP). Five runs were performed with 5,000,000 cycles each for the MCMC and a burn-in time of 10%. The value with the highest posterior probability was accepted as the best estimate.

### Phylogenetic analysis

Phylogenetic analyses of the *COII *and *ITS2 *sequences were conducted separately. Maximum parsimony (MP) and Bayesian trees were constructed for the *COII *region. For the *COII *tree, an additional 37 sequences from 32 taxa from commonly-occurring members of the genus *Anopheles *in the Australasian and Indo-Malayan regions were included (almost all are identified in [5]; [additional file 1]). We rooted the trees with *Bironella hollandi *and *Drosophila melanogaster*. Sequences were aligned using the ClustalW option of MEGA, under default parameters, and obvious alignment errors were edited by eye. The general time-reversible model with a proportion of invariable sites and a rate variation among sites following a gamma distribution (GTR+I+G; [53,54]) was selected as the best-fit model of nucleotide substitutions for the *COII *dataset to use in the Bayesian analysis from MODELTEST version 3.7 [55]. Bayesian posterior probabilities were calculated using a Metropolis-coupled Markov chain Monte Carlo (MCMC) sampling approach in MRBAYES [56,57]. Four simultaneous chains were run for 5,000,000 generations in two independent runs with trees sampled every 100 generations. After this number of generations, the standard deviation of split frequencies dropped almost to zero (< 0.0001) indicating that the run had become stationary and a sufficient sample from the posterior probability distribution had been obtained. After a burn-in of the first 25% of trees, a consensus tree was constructed from the remaining 75,002 trees with > 50% posterior probability. The MP trees were constructed using a heuristic search (1000 random addition searches) and tree-bisection-reconstruction (TBR) branch swapping, saving 100 trees per replicate in PAUP* 4.0b10 [58]. All trees were summarized into a strict consensus tree. One thousand bootstrap replicates were generated with the heuristic search option with 10 random additional searches per replicate. For both phylogenetic analyses, indels in alignments were considered as missing data.

It was not possible to include all species from COII for *ITS2*, because not all representative sequences were available in GenBank. Also, some sequences of distant taxa were too divergent from the *An. punctulatus *group to be aligned unambiguously. However, representative sequences from different parts of Australia, Papua New Guinea and Vanuatu for *An. farauti s.s*. and two different sequences for *An. irenicus *were available in GenBank. So the *ITS2 *dataset was finally constructed with 24 additional sequences (obtained from GenBank [additional file 1]) representing the *An. punctulatus *group, rendering *An. koliensis *the only outgroup. MP and Bayesian analyses were performed for *ITS2 *sequences to reconstruct phylogenetic trees using the same methodology as implemented for *COII *analysis. The Hasegawa, Kishino, Yano 85 [52] with rate variation among sites following a gamma distribution (HKY85 + G) was found to be the best-fit model for the *ITS2 *data and was selected for phylogenetic analyses. Again indels in alignments were considered as missing data. These trees were outgrouped with *An. koliensis *[5,7].

### Population genetic structure and gene flow

The extent of short-term genetic distance and the level of gene flow between groups were estimated by pairwise *F*_ST _values [59] and tested for significance by 110 permutations. The levels of gene flow (*Nm*) were estimated as an absolute number of migrants exchanged between the two populations from these *F*_ST _values [60]. In addition, divergence within population groups was estimated by an approach based on Nei & Li [61]. Also, the population genetic structure was calculated by analyzing molecular variance (AMOVA, [62]) in ARLEQUIN. AMOVA takes into account the number of molecular differences between haplotypes. Statistical significance was tested by a non-parametric permutation approach [62]. AMOVA estimates genetic variations as genetic differentiation among groups (Φ_CT_), among populations within groups (Φ_SC_) and among populations relative to total samples (Φ_ST_).

## Authors' contributions

AUH participated in the design of the study, performed the statistical analysis and wrote the manuscript. SS collected the samples, participated in the design and coordination of the study, and helped to draft the manuscript. CF, RLI and MH advised on the analysis and helped to draft the manuscript. MK, HB and BA planned and collected the samples. All authors approved the final manuscript.

## Supplementary Material

Additional file 1 Table of species, taxonomic grouping (genus, subgenus, series, group/complex), sampling localities, personal voucher numbers or references, GenBank accession number and haplotypes. Click here for file

Additional file 2 Sampling sites, number of samples and haplotype distribution in different populations for *An. farauti s.s. *and *An. irenicus *on Guadalcanal and Malaita Islands for mitochondrial *COII *data. Click here for file

Additional file 3 Population mismatch distribution among mitochondrial *COII *haplotypes for *An. farauti s.s*. and *An. irenicus *on Guadalcanal and Malaita Islands, grouped by geographical region. Click here for file

Additional file 4 Pairwise genetic distance of *An. irenicus *(*A. i.*) with *An. farauti s.s. *(*A. f.*) of Guadalcanal (GI), Malaita (MI), Papua New Guinea (PNG) and Vanuatu (Van) based on mitochondrial *COII *data. Click here for file

Additional file 5 Analysis of molecular variance (AMOVA) for *An. farauti s.s*. and *An. irenicus *on Guadalcanal and Malaita Islands based on mitochondrial *COII *data, grouped by geographical region. Click here for file
