# Supplementary material for: Genetic diversity in two sibling species of the Anopheles punctulatus group of mosquitoes on Guadalcanal in the Solomon Islands
Source: BMC Evol Biol. 2008 Nov 24;8:318. doi: 10.1186/1471-2148-8-318 (PMC2612007; doi:10.1186/1471-2148-8-318)
Supplement: Additional file 1 — Table of species, taxonomic grouping (genus, subgenus, series, group/complex), sampling localities, personal voucher numbers or references, GenBank accession number and haplotypes. [file 1471-2148-8-318-S1.doc]

### Additional file 1 – Table of species, taxonomic grouping (genus, subgenus, series, group/complex), sampling localities, personal voucher numbers or references, GenBank accession number and haplotypes

| Taxonomic grouping  (genus, subgenus, series, group/complex) | Species | Sampling locality | Voucher/ reference | GenBank  *COII* (*ITS2*) | Haplotype  *COII* (*ITS2*) |
| --- | --- | --- | --- | --- | --- |
| *Anopheles*, *Anopheles*, Myzorhynchus Series | *An. bancroftii* | Australia | *a* | U94290 |  |
|  | *An. quadrimaculatus* | Orlando strain | *b* | L04272 |  |
| *Anopheles*, *Cellia*, Myzomyia Series | *An. flavirostris* | Philippines | *a* | U94300 |  |
|  | *An. filipinea* | Philippines | *a* | U94299 |  |
|  | *An. mangyanus* | Philippines | *a* | U94309 |  |
| *Anopheles*, *Cellia*, Pyretophorus Series | *An. gambiae* | G3 strain | *b* | L20934 |  |
|  | *An. subpictus* | Philippines | *a* | U94314 |  |
|  | *An. litoralis* | Philippines | *a* | U94305 |  |
| *Anopheles*, *Cellia*, Neocellia Series | *An. karwari* | PNG | *a* | U94302 |  |
|  | *An. annularis* | Philippines | *a* | U94284 |  |
| *Anopheles*, *Cellia*, Neomyzomyia Series | *An. tessellatus* | Philippines | *a* | U94315 |  |
|  | *An. kochi* | Philippines | *a* | U94303 |  |
|  | *An. balabacensis* | Philippines | *a* | U94289 |  |
|  | *An. longirostris* | PNG | *a* | U94306 |  |
|  | *An. hilli* | Australia | *a* | U94301 |  |
|  | *An. amictus* | Australia | *a* | U94283 |  |
|  | *An. meraukensis* | Australia | *a* | U94310 |  |
|  | *An. novaguinensis* | Australia | *a* | U94311 |  |
| *Annulipes* Complex | *An. annulipes* sp. A | Australia | *a* | U94285 |  |
|  | *An. annulipes* sp. B | Australia | *a* | U94286 |  |
|  | *An. annulipes* sp. D | Australia | *a* | U94287 |  |
|  | *An. annulipes* sp. G | Australia | *a* | U94288 |  |
| *Lungae* Complex | *An. lungae* | SI, Malaita | GL0105, *^a^* | U94307 |  |
|  | *An. solomonis* | SI, Malaita | TL0504, *^a^* | U94308 |  |
| *Punctulatus* Group | *An. farauti s.s.* | SI, Guadalcanal  (Tamboko 1) | TAA001 | DQ674709  (EF619441) | S1 (S1) |
|  |  |  | TAA009 | DQ674709 | S1 |
|  |  |  | TAA012 | DQ674709 | S1 |
|  |  |  | TAB001 | DQ674709 | S1 |
|  |  |  | TAB005 | DQ674709 | S1 |
|  |  |  | TAB008 | DQ674709 | S1 |
|  |  |  | TAB009 | DQ674709 | S1 |
|  |  |  | TAB010 | DQ674709 | S1 |
|  |  |  | TAB011 | DQ674709 | S1 |
|  |  |  | TAC003 | DQ674709 | S1 |
|  |  |  | TAC005 | DQ674709 | S1 |
|  |  |  | TAC006 | DQ674709 | S1 |
|  |  |  | TAC010 | DQ674709 (EF619441) | S1 (S1) |
|  |  |  | TAD003 | DQ674709 | S1 |
|  |  |  | TAD004 | DQ674709 | S1 |
|  |  |  | TAA002 | DQ674710 (EF619441) | S2 (S1) |
|  |  |  | TAA003 | DQ674710 | S2 |
|  |  |  | TAA004 | DQ674710 | S2 |
|  |  |  | TAA005 | DQ674710 | S2 |
|  |  |  | TAA006 | DQ674710 | S2 |
|  |  |  | TAA011 | DQ674710 | S2 |
|  |  |  | TAC007 | DQ674710 | S2 |
|  |  |  | TAA08 | DQ674711 (EF619441) | S3 (S1) |
|  |  |  | TAA014 | DQ674712 | S4 |
|  |  |  | TAB006 | DQ674713 | S5 |
|  |  |  | TAB007 | DQ674714 | S6 |
|  |  |  | TAC004 | DQ674715 | S7 |
|  |  |  | TAC008 | DQ674716 (EF619441) | S8 (S1) |
|  |  |  | TAC009 | DQ674717 | S9 |
|  |  |  | TAD002 | DQ674718 | S10 |
|  |  |  | TAD005 | DQ674719 | S11 |
|  |  |  | TAD006 | DQ674720 | S12 |
|  |  |  | TAD007 | DQ674721 | S13 |
|  |  | SI, Guadalcanal  (Tamboko 2) | TL0604 | DQ674709 (EF619441) | S1 (S1) |
|  |  |  | TL1107 | DQ674709 | S1 |
|  |  |  | TL1110 | DQ674709 | S1 |
|  |  |  | TL1115 | DQ674709 | S1 |
|  |  |  | TL1902 | DQ674709 | S1 |
|  |  |  | TL1111 | DQ674710 (EF619441) | S2 (S1) |
|  |  |  | TL1113 | DQ674710 | S2 |
|  |  |  | TL1901 | DQ674710 | S2 |
|  |  |  | TL1206 | DQ674719 | S11 |
|  |  |  | TL1112 | DQ674723 | S15 |
|  |  | SI, Guadalcanal | TAE002 | DQ674709 | S1 |
|  |  | (Tavavao) | TAE004 | DQ674709 | S1 |
|  |  |  | TAF003 | DQ674709 | S1 |
|  |  |  | TAF004 | DQ674709 | S1 |
|  |  |  | TAG002 | DQ674709 | S1 |
|  |  |  | TAG003 | DQ674709 | S1 |
|  |  |  | TAG005 | DQ674709 | S1 |
|  |  |  | TAG006 | DQ674709 | S1 |
|  |  |  | TAE001 | DQ674710 | S2 |
|  |  |  | TAE003 | DQ674710 | S2 |
|  |  |  | TAE005 | DQ674710 | S2 |
|  |  |  | TAF002 | DQ674710 | S2 |
|  |  |  | TAF005 | DQ674710 | S2 |
|  |  |  | TAF006 | DQ674710 | S2 |
|  |  |  | TAG001 | DQ674710 | S2 |
|  |  |  | TAG004 | DQ674710 | S2 |
|  |  |  | TAE006 | DQ674720 | S12 |
|  |  |  | TAF001 | DQ674722 | S14 |
|  |  | SI, Guadalcanal  (Komimbo) | ML0514 | DQ674709 (EF619441) | S1 (S1) |
|  |  |  | ML0502 | DQ674710 (EF619441) | S2 (S1) |
|  |  |  | ML0512 | DQ674710 (EF619441) | S2 (S1) |
|  |  |  | ML0511 | DQ674720 (EF619441) | S12 (S1) |
|  |  |  | ML0503 | EF619417 | S20 |
|  |  |  | ML0504 | EF619418 | S21 |
|  |  |  | ML0505 | EF619419 | S22 |
|  |  |  | ML0506 | EF619420 | S23 |
|  |  |  | ML0508 | EF619421 | S24 |
|  |  |  | ML0510 | EF619422 | S25 |
|  |  | SI, Guadalcanal  (Sopapera) | SL0201 | DQ674709 (EF619441) | S1 (S1) |
|  |  |  | SL0204 | DQ674709 | S1 |
|  |  | SI, Guadalcanal (Koli) | LL4B06 | DQ674709 | S1 |
|  |  |  | LL4B01 | DQ674710 (EF619441) | S2 (S1) |
|  |  |  | LL4B03 | DQ674710 (EF619441) | S2 (S1) |
|  |  |  | LL4B05 | DQ674710 | S2 |
|  |  |  | LL4B02 | EF619416 | S19 |
|  |  | SI, Guadalcanal | *c* | (AF104317) | (GI) |
|  |  | SI, Malaita (Fiu) | FL1408 | DQ674709 (EF619441) | S1 (S1) |
|  |  |  | FL1410 | DQ674709 | S1 |
|  |  |  | FL1415 | DQ674709 | S1 |
|  |  |  | FL1418 | DQ674714 (EF619441) | S6 (S1) |
|  |  |  | FL1306 | DQ674724 (EF619441) | S16 (S1) |
|  |  |  | FL1307 | DQ674724 | S16 |
|  |  |  | FL1409 | DQ674725 | S17 |
|  |  |  | FL1416 | DQ674726 | S18 |
|  |  | SI, Malaita (Mawa) | AL0504 | DQ674709 (EF619441) | S1 (S1) |
|  |  |  | AL0508 | DQ674709 | S1 |
|  |  |  | AL0509 | DQ674709 | S1 |
|  |  |  | AL0515 | DQ674713 | S5 |
|  |  |  | AL0507 | EF619417 | S20 |
|  |  |  | AL0503 | EF619423 (EF619441) | S26 (S1) |
|  |  | Vanuatu | *a* | U94292 | Van |
|  |  |  | *c* | (AF104315) | (Van) |
|  |  | PNG | DAA034 | DQ674728 | PNG1 |
|  |  |  | DAB016 | DQ674729 | PNG2 |
|  |  | PNG, Northern part | *c* | (AF104326) | (PNG N1) |
|  |  |  | *c* | (AF104325) | (PNG N2) |
|  |  |  | *c* | (AF104324) | (PNG N3) |
|  |  |  | *c* | (AF104323) | (PNG N4) |
|  |  |  | *c* | (AF104321) | (PNG N5) |
|  |  |  | *c* | (AF104319) | (PNG N6) |
|  |  | PNG, Southern part | *c* | (AF104322) | (PNG S1) |
|  |  | PNG, Rabaul | *c* | (AF104320) | (Rab 1) |
|  |  |  | *d* | (AF030406) | (Rab 2) |
|  |  | Australia, Northern territory | *c* | (AF104316) | (AUS N1) |
|  |  |  | *c* | (AF104314) | (AUS N2) |
|  |  |  | *e* | (AF055984) | (AUS N3) |
|  |  | Australia, Queensland | *c* | (AF104318) | (AUS Q1) |
|  | *An. hinesorum* | SI | TL1001 | EF619425 | SI1 |
|  |  |  | FL1203 | EF619429 | SI2 |
|  |  | PNG | WF2011 | *f* | PNG |
|  |  | Australia, Northern territory | *e* | (AF033213) |  |
|  | *An. torresiensis* | Australia | *a* | U94294 |  |
|  |  | Australia, Northern territory | *e* | (AF033214) |  |
|  | *An. farauti* 4 | PNG | *a* | U94295 |  |
|  |  |  | *e* | (AF033215) |  |
|  | *An. farauti* 5 | PNG | *a* | U94296 |  |
|  |  |  | *e* | (AF033216) |  |
|  | *An. farauti* 6 | PNG | *a* | U94297 |  |
|  |  |  | *e* | (AF033217) |  |
|  | *An. irenicus* | SI, Guadalcanal  (Tamboko 2) | TL0201 | DQ984210  (EF619442) | I1 (I1) |
|  |  |  | TL0202 | DQ984210 (EF619442) | I1 (I1) |
|  |  |  | TL0203 | DQ984210 | I1 |
|  |  |  | TL0205 | DQ984210 | I1 |
|  |  |  | TL0601 | DQ984210 | I1 |
|  |  |  | TL1002 | DQ984210 | I1 |
|  |  |  | TL1108 | DQ984210 | I1 |
|  |  |  | TL1109 | DQ984210 | I1 |
|  |  |  | TL1114 | DQ984210 | I1 |
|  |  |  | TL1202 | DQ984210 | I1 |
|  |  |  | TL1205 | DQ984210 | I1 |
|  |  |  | TL1304 | DQ984210 | I1 |
|  |  |  | TL1305 | DQ984210 | I1 |
|  |  |  | TL1906 | DQ984210 | I1 |
|  |  |  | TL2010 | DQ984210 | I1 |
|  |  |  | TL0603 | DQ984212 (EF619442) | I3 (I1) |
|  |  |  | TL1106 | DQ984212 | I3 |
|  |  |  | TL1904 | DQ984213 | I4 |
|  |  |  | TL1905 | DQ984214 | I5 |
|  |  |  | TL2004 | DQ984215 | I6 |
|  |  |  | TL2005 | DQ984216 | I7 |
|  |  | SI, Guadalcanal | MLB001 | DQ984210 | I1 |
|  |  | (Komimbo) | MLB002 | DQ984210 | I1 |
|  |  |  | MLB004 | DQ984211 | I2 |
|  |  | SI, Guadalcanal  (Sopapera) | SL1A02 | DQ984210 (EF619442) | I1 (I1) |
|  |  |  | SL1A12 | DQ984210 | I1 |
|  |  |  | SL1A14 | DQ984210 | I1 |
|  |  |  | SL1B06 | DQ984210 | I1 |
|  |  |  | SL1B08 | DQ984210 | I1 |
|  |  |  | SL1B15 | DQ984210 | I1 |
|  |  |  | SL1B16 | DQ984210 | I1 |
|  |  |  | SL0205 | DQ984210 | I1 |
|  |  |  | SL1A08 | EF619435 (EF619442) | I8 (I1) |
|  |  |  | SL1A20 | EF619435 | I8 |
|  |  |  | SL1A13 | EF619436 (EF619442) | I9 (I1) |
|  |  |  | SL1A19 | EF619436 | I9 |
|  |  |  | SL1B01 | EF619436 | I9 |
|  |  |  | SL1B02 | EF619437 | I10 |
|  |  |  | SL1B05 | EF619437 | I10 |
|  |  |  | SL1B17 | EF619437 | I10 |
|  |  |  | SL0203 | EF619438 | I11 |
|  |  |  | SL0206 | EF619439 | I12 |
|  |  | SI, Guadalcanal (Patima) | PL0308 | EF619440 (EF619442) | I13 (I1) |
|  |  | SI, Guadalcanal | *e* | (AF033218) | (G1) |
|  |  |  | *g* | (EF042725) | (G2) |
|  | *An. koliensis* | PNG | *a* | U94304 |  |
|  |  | PNG, Sepik province | *e* | (AF033219) |  |
|  | *An. punctulatus* | PNG | *a* | U94312 |  |
|  |  | PNG, Sepik province | *e* | (AF033220) |  |
|  | *An.* sp nr *puntulatus* | Irian Jaya | *a* | U94313 |  |
|  |  | PNG | *e* | (AF033221) |  |
| *Bironella* | *Bi. hollandi* | SI, Guadalcanal | TL0304 | EU477542 |  |
| *Drosophila* | *D. melanogaster* | - | *b* | U37541 |  |

*^a^* Foley DH, Bryan JH, Yeates D, Saul A: **Evolution and systematics of Anopheles: insights from a molecular phylogeny of Australasian mosquitoes.** *Molecular phylogenetics and evolution* 1998, **9:**262-275.

*^b^* From GenBank.

*^c^* Beebe NW, Cooper RD, Foley DH, Ellis JT: **Populations of the south-west Pacific malaria vector Anopheles farauti s.s. revealed by ribosomal DNA transcribed spacer polymorphisms.** *Heredity* 2000, **84 ( Pt 2):**244-253.

*^d^* Beebe et al., 1998 From GenBank

*^e^* Beebe NW, Ellis JT, Cooper RD, Saul A: **DNA sequence analysis of the ribosomal DNA ITS2 region for the Anopheles punctulatus group of mosquitoes**. *Insect Mol. Biol.* 1999, **8:** 381-390.

*^f^* Fujimoto et al., unpublished data.

*^g^* Bower et al., 2006 From GenBank.
