# Supplementary material for: Genetic diversity in two sibling species of the Anopheles punctulatus group of mosquitoes on Guadalcanal in the Solomon Islands
Source: BMC Evol Biol. 2008 Nov 24;8:318. doi: 10.1186/1471-2148-8-318 (PMC2612007; doi:10.1186/1471-2148-8-318)
Supplement: Additional file 2 — Sampling sites, number of samples and haplotype distribution in different populations for An. farauti s.s. and An. irenicus on Guadalcanal and Malaita Islands for mitochondrial COII data. [file 1471-2148-8-318-S2.doc]

### Additional file 2 – Sampling sites, number of samples and haplotype distribution in different populations for *An. farauti s.s.* and *An. irenicus* on Guadalcanal and Malaita Islands for mitochondrial *COII* data.

***N* and *S* are the number of sequences and number of segregating sites, respectively. Bold numbers indicate haplotypes that occurred in more than one site. Number of samples is given in parentheses.**

|  | *N* | *S* | Haplotype observed | Haplotype distribution |
| --- | --- | --- | --- | --- |
| *Anopheles farauti s.s.* |  |  |  |  |
| Overall (Guadalcanal) | 78 | 18 | 26 |  |
| Tamboko 1 (G1) | 33 | 13 | 13 | **S1** (15), **S2** (7), S3, S4, **S5**, **S6**, S7, S8, S9, S10, **S11**, **S12**, S13 |
| Tamboko 2 (G2) | 10 | 3 | 4 | **S1** (5), **S2** (3), **S11**, S15 |
| Tavavao (G3) | 18 | 4 | 4 | **S1** (8), **S2** (8), **S12**, S14 |
| Komimbo (G4) | 10 | 8 | 9 | **S1**, **S2** (2), **S12**, **S20**, S21, S22, S23, S24, S25 |
| Sopapera (G5) | 2 | 0 | 1 | **S1** (2) |
| Koli (G6) | 5 | 3 | 3 | **S1**, **S2** (3), S19 |
| Overall (Malaita) | 14 | 9 | 5 |  |
| Fiu (M1) | 8 | 6 | 5 | **S1** (3), **S6**, S16 (2), S17, S18 |
| Mawa (M2) | 6 | 4 | 4 | **S1** (3), **S5**, **S20**, S26 |
| *Anopheles irenicus* |  |  |  |  |
| Overall (Guadalcanal) | 43 | 12 | 13 |  |
| Tamboko 2 (G2) | 21 | 5 | 6 | **I1** (15), I3 (2), I4, I5, I6, I7 |
| Komimbo (G4) | 3 | 1 | 2 | **I1** (2), I2 |
| Sopapera (G5) | 18 | 6 | 3 | **I1** (8), I8 (2), I9 (3), I10 (3), I11, I12 |
| Patima (G7) | 1 | 0 | 1 | I13 |
