# Supplementary material for: Genetic diversity in two sibling species of the Anopheles punctulatus group of mosquitoes on Guadalcanal in the Solomon Islands
Source: BMC Evol Biol. 2008 Nov 24;8:318. doi: 10.1186/1471-2148-8-318 (PMC2612007; doi:10.1186/1471-2148-8-318)
Supplement: Additional file 3 — Population mismatch distribution among mitochondrial COII haplotypes for An. farauti s.s. and An. irenicus on Guadalcanal and Malaita Islands, grouped by geographical region. [file 1471-2148-8-318-S3.doc]

## Additional file 3 - Population mismatch distribution among mitochondrial COII haplotypes for An. farauti s.s. and An. irenicus on Guadalcanal and Malaita Islands, grouped by geographical region.
