# Supplementary material for: Genetic diversity in two sibling species of the Anopheles punctulatus group of mosquitoes on Guadalcanal in the Solomon Islands
Source: BMC Evol Biol. 2008 Nov 24;8:318. doi: 10.1186/1471-2148-8-318 (PMC2612007; doi:10.1186/1471-2148-8-318)
Supplement: Additional file 4 — Pairwise genetic distance of An. irenicus (A. i.) with An. farauti s.s. (A. f.) of Guadalcanal (GI), Malaita (MI), Papua New Guinea (PNG) and Vanuatu (Van) based on mitochondrial COII data. [file 1471-2148-8-318-S4.doc]

### Additional file 4 – Pairwise genetic distance of *An. irenicus* (*A. i.*) with *An. farauti s.s.* (*A. f.*) of Guadalcanal (GI), Malaita (MI), Papua New Guinea (PNG) and Vanuatu (Van) based on mitochondrial *COII* data.

Pairwise *F_ST_*, below diagonal; migration rate (*Nm*), above diagonal.

**P*≤0.05 and ***P*≤0.01; ^†^ significant test not done due to single sample.

|  | *A. f.* | | | | | *A. i.* |
| --- | --- | --- | --- | --- | --- | --- |
|  |  | GI | MI | PNG | Van | GI |
| *A. f.* | GI |  | 3.296 | 0.063 | 0.123 | 0.065 |
|  | MI | 0.132** |  | 0.092 | 0.181 | 0.069 |
|  | PNG | 0.888** | 0.844** |  | 0.333 | 0.045 |
|  | Van | 0.802^†^ | 0.734^†^ | 0.600^†^ |  | 0.054 |
| *A. i.* | GI | 0.885** | 0.878** | 0.918** | 0.902^†^ |  |
