# Supplementary material for: Genetic diversity in two sibling species of the Anopheles punctulatus group of mosquitoes on Guadalcanal in the Solomon Islands
Source: BMC Evol Biol. 2008 Nov 24;8:318. doi: 10.1186/1471-2148-8-318 (PMC2612007; doi:10.1186/1471-2148-8-318)
Supplement: Additional file 5 — Analysis of molecular variance (AMOVA) for An. farauti s.s. and An. irenicus on Guadalcanal and Malaita Islands based on mitochondrial COII data, grouped by geographical region. [file 1471-2148-8-318-S5.doc]

### Additional file 5 – Analysis of molecular variance (AMOVA) for *An. farauti s.s.* and *An. irenicus* on a) Guadalcanal and Malaita Islands and b) Guadalcanal Island based on mitochondrial *COII* data, grouped by geographical region.

*^ns^P*>0.05, **P*≤0.05 and ***P*≤0.01

| Variance component | Percentage of variation | Φ statistics |
| --- | --- | --- |
| a) Guadalcanal and Malaita Islands |  |  |
| Among groups | 86.26 | Φ_CT_ = 0.863** |
| Among populations within group | 0.20 | Φ_SC_ = 0.014 *^ns^* |
| Within populations | 13.55 | Φ_ST_ = 0.865** |
|  |  |  |
| b) Guadalcanal Island |  |  |
| Among groups | 88.57 | Φ_CT_ = 0.886** |
| Among populations within group | 0.38 | Φ_SC_ = 0.033 *^ns^* |
| Within populations | 11.06 | Φ_ST_ = 0.889** |
